# Supplementary material for: A new scheduling method based on sequential time windows developed to distribute first-aid medicine for emergency logistics following an earthquake
Source: PLoS One. 2021 Feb 23;16(2):e0247566. doi: 10.1371/journal.pone.0247566 (PMC7901742; doi:10.1371/journal.pone.0247566)
Supplement: Attachment — Submitted filename: response to the comments.doc [file pone.0247566.s001.doc]

**response to the comments**

**(1)Editor Comments:**Our editorial staff has assessed your submission, and we have significant concerns about the grammar, usage, and overall readability of the manuscript. We believe that the manuscript is much more likely to be accepted if it is easy for the reviewers to read and understand. We therefore request that you revise the text to fix the grammatical errors and improve the overall readability of the text before we send it for review.

**Author response:** We asked a professional language editing company AJE to polish the language. And we submitted the certificate and editing version as supporting information.

**(2)Editor Comments:**There are many reference errors in the paper. Please fix the errors and then resubmit the paper.

**Author response:** We have revised all the reference errors.
