# Supplementary material for: A new scheduling method based on sequential time windows developed to distribute first-aid medicine for emergency logistics following an earthquake
Source: PLoS One. 2021 Feb 23;16(2):e0247566. doi: 10.1371/journal.pone.0247566 (PMC7901742; doi:10.1371/journal.pone.0247566)
Supplement: Attachment — Submitted filename: Response to Reviewers.doc [file pone.0247566.s002.doc]

**response to the comments**

**(1)Reviewer #1 Comments:** Paper is good but needs to be improved. In each chapter, in addition to the summary, results and conclusion, there should be references. References are placed in the paper only in the Literature review chapter. It is necessary to add references to other chapters, in order to improve the model you used in the literature paper. Insert a chapter on discussions, because you have a lot of simulations, explain them, and connect them with the previous papers.

**Author response:** In each chapter, I have added some literature related to the content of my article, and these literature can help to better understand my article. I inserted a chapter on simulation and related them to the previous paper, and I also added some content to help readers better understand the simulation process.

**(2)Reviewer #2 Comments:**The paper has potential for publication, but should be in some parts improved. Generally, paper deserves attention because deals with very important topic. Also, applied methodology is good.

Please improve your paper adopting the following suggestions:

- Introduction section should be more clear and precise. Please write clear aims and contributions.

- As the last paragraph in introduction please add short structure of the whole paper.

- Please consider and cite the following new references:

Biswas, T. K., & Das, M. C. (2020). Selection of the barriers of supply chain management in Indian manufacturing sectors due to COVID-19 impacts. Operational Research in Engineering Sciences: Theory and Applications, 3(3), 1-12.

Adhikary, K., Roy, J., & Kar, S. (2019). Newsboy problem with birandom demand. Decision Making: Applications in Management and Engineering, 2(1), 1-12.

Giri, B. C., & Dey, S. (2020). Game theoretic models for a closed-loop supply chain with stochastic demand and backup supplier under dual channel recycling. Decision Making: Applications in Management and Engineering, 3(1), 108-125.

Write guidelines for future research in conclusion.

**Author response:** I revised the introduction to make it more clear and precise, and wrote contributions and and aims to help the reader better understand. I added short structure of the whole paper in the last paragraph in introduction. After reading the recommended references, I thought it is helpful to cite these references in my article for readers. At last, I wrote guidelines for future research in conclusion.
